# Modeling the cost-effectiveness of insect rearing on artificial diets: A test with a tephritid fly used in the sterile insect technique

**DOI:** 10.1371/journal.pone.0173205

**Published:** 2017-03-03

**Authors:** Carlos Pascacio-Villafán, Andrea Birke, Trevor Williams, Martín Aluja

**Affiliations:** Red de Manejo Biorracional de Plagas y Vectores, Clúster Científico y Tecnológico BioMimic^®^, Instituto de Ecología, A.C., Xalapa, Veracruz, Mexico; International Atomic Energy Agency, AUSTRIA

## Abstract

We modeled the cost-effectiveness of rearing *Anastrepha ludens*, a major fruit fly pest currently mass reared for sterilization and release in pest control programs implementing the sterile insect technique (SIT). An optimization model was generated by combining response surface models of artificial diet cost savings with models of *A*. *ludens* pupation, pupal weight, larval development time and adult emergence as a function of mixtures of yeast, a costly ingredient, with corn flour and corncob fractions in the diet. Our model revealed several yeast-reduced mixtures that could be used to prepare diets that were considerably cheaper than a standard diet used for mass rearing. Models predicted a similar production of insects (pupation and adult emergence), with statistically similar pupal weights and larval development times between yeast-reduced diets and the standard mass rearing diet formulation. Annual savings from using the modified diets could be up to 5.9% of the annual cost of yeast, corn flour and corncob fractions used in the standard diet, representing a potential saving of US $27.45 per ton of diet (US $47,496 in the case of the mean annual production of 1,730.29 tons of artificial diet in the Moscafrut mass rearing facility at Metapa, Chiapas, Mexico). Implementation of the yeast-reduced diet on an experimental scale at mass rearing facilities is still required to confirm the suitability of new mixtures of artificial diet for rearing *A*. *ludens* for use in SIT. This should include the examination of critical quality control parameters of flies such as adult flight ability, starvation resistance and male sexual competitiveness across various generations. The method used here could be useful for improving the cost-effectiveness of invertebrate or vertebrate mass rearing diets worldwide.

## Introduction

Artificial diets are foods synthesized from one or more ingredients that may be completely defined chemically, partially defined or not defined [1]. Artificial diets are used for the domestication, colonization, mass production and maintenance of a large number of animal species important for human welfare. For instance, fishes [2], crustaceans [3], mollusks [4], echinoderms [5], pork [6], poultry [7] and insects [1] are reared and maintained on different types of artificial diets. Artificial diets must fulfill sensory requirements and be nutritious for animals within a framework of economic feasibility [1]. In reality, the production of artificial diets is one of the most substantial direct input costs in many areas related to animal breeding [8, 9, 6].

Several rearing programs have benefited from the application of mixture experiments and response surface methods (RSM) as a strategy for artificial diet optimization [10–13]. This strategy of experimentation allows for the construction of empirical models that are useful for the simultaneous prediction and optimization of multiple responses [14, 15]. This experimental approach and modeling method provide results that are readily applicable, improve the efficacy of resource use and diminish the risks of inference in decision making in artificial diet research and development [16].

In this study, our goal was to develop a practical approach that could be implemented in any animal rearing facility that uses artificial diets to lower costs without compromising quality. To reach this goal, we used a mixture experiment and RSM approaches to model the cost-effectiveness of rearing a major fruit fly pest, the Mexican fruit fly, *Anastrepha ludens* (Loew) (Diptera: Tephritidae), on artificial diet. Millions of artificially-reared *A*. *ludens* are produced and sterilized on a daily basis at the Moscafrut facility of the National Fruit Fly Program SENASICA-SAGARPA Mexico, at Metapa, Chiapas, Mexico [17]. These insects are employed in pest management programs based on the Sterile Insect Technique (SIT), involving the release of massive numbers of sterile male flies [17, 18]. There are more than 20 integrated pest management programs across the world using the SIT to control fruit flies [19]. Due to the success of the technique, new rearing facilities are being constructed in many countries [20, 21].

Mass production of sterile flies depends on the use of artificial diets, which account for about 30% of the total cost of production of *A*. *ludens* at Moscafrut [22]. One of the most expensive ingredients in the artificial diet of *A*. *ludens* is dried yeast, which has a 45% protein content [23, 24]. The results of a previous study indicated that large amounts of protein in *A*. *ludens* artificial diet may not be fully utilized by larvae and may thus be unnecessary [23]. In the present study, we hypothesized that the yeast content of the mass rearing artificial diet of *A*. *ludens* could be significantly reduced and substituted by cheaper ingredients to reduce the costs of *A*. *ludens* mass rearing for use in SIT. We predicted that by reducing the yeast content in a mixture with cheaper corn flour and corncob fractions, we could identify cheaper mixtures of these ingredients in the diet of *A*. *ludens* that would allow the production of similar numbers of flies with similar larval development times and pupal weights to the flies produced on the standard artificial diet used for mass production.

## Materials and methods

### Experimental insects

*Anastrepha ludens* were obtained from the Red de Manejo Biorracional de Plagas y Vectores of the Instituto de Ecología, A.C., in Xalapa, Veracruz state, Mexico. This colony of *A*. *ludens* was started in 1998. It has been maintained on artificial diets for over 120 generations with occasional introductions of wild flies from naturally-infested citrus fruit collected from commercial orchards in Veracruz state, Mexico [24]. The rearing process used for *A*. *ludens* is described elsewhere [25]. In brief, ca. 3000 adult flies aged 13–16 days were kept in plexiglass cages (30 × 30 × 60 cm) with *ad libitum* access to water and food (3:1 sugar: hydrolyzed protein). Flies oviposited on transparent silicon media. Eggs were collected from oviposition media and washed in 0.2% (wt/vol) sodium benzoate solution, then rinsed with tap water, placed on pieces of terylene cloth on top of moistened cotton inside Petri dishes, and incubated in a dark room at 30 ± 1°C and 70 ± 5% relative humidity (hereafter incubation room) for four days until they hatched. We collected eggs from four plexiglass cages as described before, and on the day of hatching, one cohort of larvae was used in the experiments.

### Artificial diets

Experimental diets were based on a standard diet formulation used for mass production of *A*. *ludens* [17, 23]. All diets consisted of constant levels of cane sugar, citric acid, guar gum, preservatives and water that were combined with various mixtures of inactive dried yeast (*Candida utilis*), corn flour and corncob fractions (hereafter yeast: corn flour: corncob fractions mixtures) (Table 1).

**Table 1:** The experimental diet mixtures tested.

Mixture No.^a^	Yeast (%)	Corn flour (%)	Corncob fractions (%)
1 (standard)	6.0	5.3	19.0
2	5.0	5.3	20.0
3	5.0	5.8	19.5
4	5.0	6.3	19.0
5	4.5	5.3	20.5
6	4.5	6.8	19.0
7	4.3	5.7	20.3
8	4.3	6.5	19.5
9	4.0	5.3	21.0
10	4.0	6.3	20.0
11	4.0	7.3	19.0
12	3.5	5.8	21.0
13	3.5	6.5	20.3
14	3.5	7.3	19.5
15	3.0	5.3	22.0
16	3.0	6.3	21.0
17	3.0	6.8	20.5
18	3.0	7.3	20.0
19	3.0	8.3	19.0

^a^ Mixtures were used to prepare artificial diets that had sugar (8.2%), sodium benzoate (0.4%), methylparaben (0.1%), citric acid (0.44%), guar gum (0.1%) and water (60.46%). Mixture No. 1 represents the standard mass rearing diet formulation.

### Diet mixture experiment

**Fig 1:**
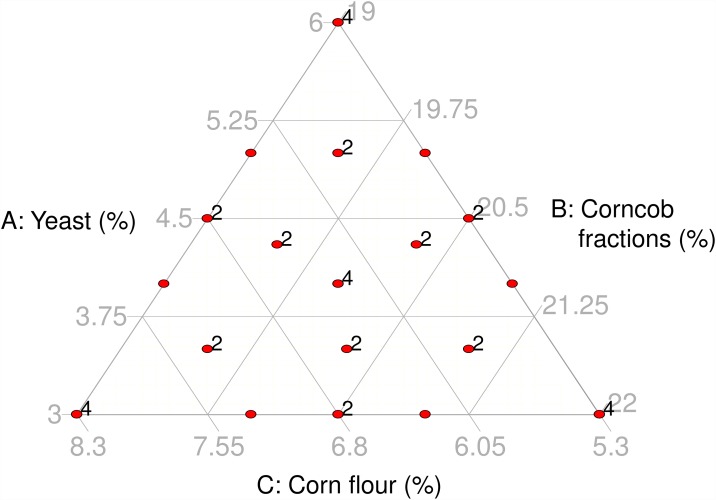
Three-component mixture experiment design space employed to model the cost-effectiveness of rearing *Anastrepha ludens* on artificial diet. The design space was constrained by 3% ≤ yeast ≤ 6%, 5.3% ≤ corn flour ≤ 8.3%, and 19% ≤ corncob fractions ≤ 22%. The red points indicate the coordinates of the mixtures evaluated, and the number next to some points, indicate the number of replicates of those mixtures. The mixture consisting of 6% yeast, 5.3% corn flour and 19% corncob fractions (red point at the top vertex), represents the standard mass rearing mixture.

We used a three-component mixture experiment [26]. Mixture components were yeast, corn flour and corncob fractions. Yeast was the target ingredient to reduce in the mixture. The proportion of each component in the design space ranged as follows: 3% ≤ yeast ≤ 6%, 5.3% ≤ corn flour ≤ 8.3% and 19% ≤ corncob fractions ≤ 22% (Fig 1). All possible mixtures in the design space (Fig 1) made up 30.3% (by weight) of the whole diet, i.e., 100% of the artificial diet. The remaining 69.7% of the diet comprised the same ingredients mentioned above in the ‘Artificial diets’ section (i.e. cane sugar, citric acid, guar gum, preservatives and water; Table 1). The mixture in the top vertex of the design space (6% yeast, 5.3% corn flour and 19% corncob fractions, Fig 1) represents the standard mass rearing diet. The experiment consisted of 19 yeast: corn flour: corncob fractions diet mixtures (Table 1), some of which were replicated two or four times (Fig 1), for 40 experimental runs (S1 Table). An experimental run is a unit operation with a specified factor level combination (i.e., a specific mixture) that produces measured responses [14]. The distribution of the tested mixtures in the design space (red points in Fig 1) was based on a study by Lapointe et al. [10]. The design (Fig 1) was sufficient to satisfy a cubic or higher order Scheffé polynomial response surface model, and replication of mixtures was based on attaining sufficient degrees of freedom to estimate pure error across the design space and to attain near uniform leverage for all points [27]. Run order was randomized and all runs were conducted in one block (S1 Table).

### Experimental procedure

With the exception of water, all diet ingredients required to prepare 25 g of each artificial diet were weighed on a digital balance (Ohaus TP4KD), and then hand mixed for 2 minutes in a plastic cup (7 cm diameter, 6 cm tall). Next, water was added and mixed for an additional 3 minutes. Then, a 25 g portion of each artificial diet was placed in a Petri dish (5 cm diameter, 2 cm tall) together with 25 randomly-selected neonate larvae of *A*. *ludens*. Petri dishes (without lids) with larvae were placed individually inside plastic containers (7 cm diameter, 6 cm tall) with a layer of vermiculite in the bottom and with a perforated lid to allow ventilation, and placed in the incubation room. When ready to pupate, larvae dropped from the Petri dish on to the vermiculite. Pupation was checked daily seven days after the beginning of the experiment, by sifting vermiculite on a white plastic board. Diets were also inspected for larvae pupating there. Recovered pupae were placed in plastic cups (7 cm diameter by 6 cm tall) with vermiculite, in a laboratory at 26 ± 1°C, 60 ± 5% RH and 12:12 h L:D photoperiod. Three days following pupation, pupae were weighted individually on an analytical balance (Sartorius CP64) to a precision of ± 0.1 mg, and transferred to individual cells (1.6 cm by 1.6 cm) of compartmentalized plastic dishes, covered with a transparent acrylic lid with perforations to allow ventilation, until adult emergence at 26 ± 1°C, 60 ± 5% RH and 12:12 h L:D photoperiod.

### Response variables

The following response variables were considered in the development of RSM models: **Cost (US dollars)** of each yeast: corn flour: corncob fractions mixture required for a mean annual production of 1,730.29 tons of artificial diet. This is the mean production of artificial diet at the Moscafrut mass rearing facility, calculated from diet production in the years 2013, 2014 and 2015 (Integrated Costing System Moscafrut). **Cost savings (US dollars and %)** of each mixture with respect to the cost of the mixture in a standard diet for mass rearing of *A*. *ludens* (6% yeast, 5.3% corn flour and 19% corncob fractions) required to prepare 1,730.29 tons of diet. Calculations are presented in S2 Table. **Pupation (proportion)**, expressed as the proportion of individuals that pupated in groups of 25 larvae that developed on each diet. **Duration of the larval stage (days)**, expressed as the mean time, in days, from hatching to pupation of all individuals in a diet. **Pupal weight (mg)**, expressed as the mean weight of three day-old viable pupae i.e., pupae from which adults emerged. **Adult emergence (proportion)**, expressed as the proportion of adults that emerged from pupae recovered from each diet.

### Statistical modeling

We used the Design-Expert^®^ 8 software (Stat-Ease, Inc, Minneapolis, MN) for experimental design construction, statistical modeling and all calculations. Statistical significance for all tests was set at a critical level of α = 0.05. Data on adult emergence were arcsine square root transformed before modeling to correct heteroscedasticity [28], but model parameters reported in results were back transformed to provide proportion values. All other response variables had normally distributed errors and exhibited constant variance, and were modeled without transformation.

The nature of the explanatory and response variables, and the goal of optimization, made RSM appropriate for the analyses [14]. Scheffé polynomial models, from the mean to the quartic [14, 15], were fitted sequentially to the values of each response variable. Sequential model sum of squares (Type I) was used to assess the improvement in the model fit as terms were added [14]. A lack of fit test for each model was calculated to test whether the model described the data adequately [14, 15]. The following statistics were calculated for each complete model: standard deviation, *R*^*2*^, adjusted *R*^*2*^ (*R*^*2*^_adj_), predicted *R*^*2*^ (*R*^*2*^_pred_) and predicted residual sum of squares (PRESS) [14]. Model selection was then based on: a) lack of any aliased terms, b) a low *P*-value of model terms, c) non-significant lack of fit, d) low standard deviation, e) low PRESS, f) high *R*^*2*^, *R*^*2*^_adj_ and *R*^*2*^_pred,_ and g) close agreement between *R*^*2*^_adj_ and *R*^*2*^_pred_, in relation to the other models [14].

The selected model was further evaluated by analysis of variance (ANOVA Type III). When possible, model simplification was performed by backward elimination of non-significant model terms. The relative magnitude and direction of the effects of each mixture component and their interactions on the various response variables was determined by examination of model coefficients and 95% CI. The precision of each model was examined by comparing the range of predicted values at the design points to the average variance of the prediction [14]. This statistic assesses a model’s adequacy for predictive purposes—values greater than 4.0 indicate that the model can be used for the purposes of prediction and optimization [14].

After model fitting, normality and homoscedasticity were explored graphically via normal probability plots of residuals and by plotting the internally studentized residuals versus the predicted responses [14]. Box-Cox plots were used to identify if a power law transformation could improve the fit [29]. DFFITS (a measure of influence based on the difference in fits in each predicted value) and DFBETAS (a measure of influence based on difference in model coefficients) plots were used to identify overly influential data points [30]. Externally studentized “outlier-*t*” [31, 32] and Cook’s distance [33] plots were used for examination of potential outliers. As in the case of the models fitted to data on the duration of the larval stage and pupal weight, runs 39 and 23, respectively, were identified as outliers and highly influential data points. Including these data points in the models lead to a significant lack of fit, in the case of the duration of the larval stage, and to a difference between *R*^*2*^_adj_ and *R*^*2*^_pred_ greater than 0.2, in the case of pupal weight, indicating unreliable predictions of the models [14]. Therefore, the final analysis on the duration of the larval stage and pupal weight ignored runs 39 and 23, respectively. The models including runs 39 and 23, and a report of statistic values from diagnostic plots are shown in S1 Appendix.

Finally, we used a graphical multivariate optimization technique [14] to model cost-effectiveness of *A*. *ludens* production on artificial diet. Our optimization criteria were to maximize cost savings and to maintain the average pupation, pupal weight, larval development time and adult emergence at values similar to those obtained for the standard mass rearing mixture (within a 95% confidence interval predicted for the standard mass rearing mixture). In each of the RSM models considered in the optimization analysis, the area meeting optimization criteria was highlighted from the area that did not fit the optimization criteria. Then, an overlay graph was generated, which consisted of the overlaid contour plots from each response variable. The area of operability, that is, the area meeting the optimization criteria for all responses, was highlighted from the remainder of the experimental space. We then explored the area of operability to make predictions, based on model equations, about cost savings and the response of flies to specific diet mixtures.

## Results

### Costs

**Fig 2:**
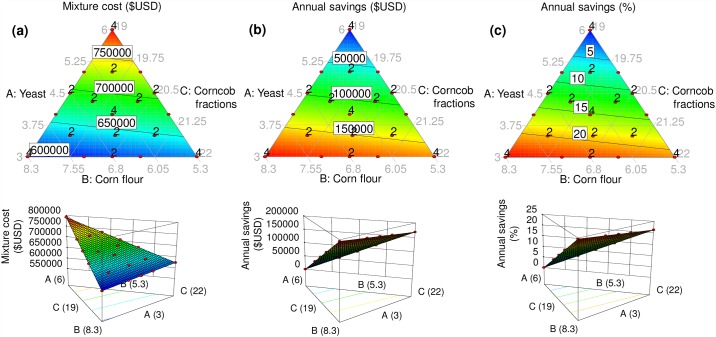
Response surface models fitted to data on diet cost. (a) Cost (US dollars), (b) cost savings (US dollars) and (c) cost savings (%) as a function of yeast: corn flour: corncob fractions mixtures in the artificial diet of *Anastrepha ludens*. Two-dimensional and three-dimensional plots for each response variable are shown in the upper and lower row, respectively.

Costs of the yeast: corn flour: corncob fractions mixtures for an estimated annual production of 1,730.29 tons of artificial diet, ranged from $791,716 in the 6:5.3:19 yeast: corn flour: corncob fractions mixture (the standard mass rearing formulation) to $596,525 in the 3:8.3:19 yeast: corn flour: corncob fractions mixture (Fig 2a). As such, the major savings accounted by the models was $195,191 (Fig 2b), which represents a saving of 24.65% of total yeast: corn flour: corncob fractions mixture used in the standard mass rearing artificial diet formulation (Fig 2c). The ingredient that contributed most to the cost of the mixture was yeast, whereas corn flour contributed most to cost savings (Tables 2 and 3, Fig 2).

**Table 2:** ANOVA, lack of fit test and summary statistics of the overall models fitted to the response variables considered in the development of RSM models.

Response variables^a^	ANOVA		R^2^	R^2^_adj_	R^2^_pred_	Adequate Precision	Model type^b^
	Model	Lack of fit					
Costs^c^	*F*_2,37_ = 6.37^7^ *P*<0.0001	-	1	1	1	-	Linear
Pupation	*F*_2,37_ = 8.52 *P* = 0.0009	*F*_16,21_ = 1.10 *P* = 0.4110	0.315	0.278	0.202	8.631	Linear
Duration of the larval stage	*F*_7,31_ = 14.83 *P*<0.0001	*F*_10,21_ = 1.56 *P* = 0.1868	0.770	0.718	0.611	13.233	Reduced special quartic
Pupal weight	*F*_4,34_ = 4.62 *P* = 0.0043	*F*_14,20_ = 1.82 *P* = 0.1069	0.352	0.276	0.116	7.242	Reduced quadratic
Adult emergence	*F*_3,36_ = 6.46 *P* = 0.0013	*F*_15,21_ = 1.60 *P* = 0.1584	0.350	0.296	0.206	8.247	Reduced quadratic

^a^ Data on adult emergence were arcsin square root transformed prior to analysis; data on all other response variables were modeled without transformation as analysis of residuals and a Box-Cox plot analysis did not suggested the need for transformation.

^b^ Model reduction was performed by backward elimination of non-significant model terms with α = 0.05 for terms be removed from the model.

^c^ Represents the ANOVA and summary statistics of models fitted to data on cost (US dollars) and cost savings (US dollars and %).

**Table 3:** Effects of yeast (A), corn flour (B) and corncob fractions (C) mixtures on the response variables considered in the development of RSM models.

Effects	*F*-value^a^	Coefficient estimate^b^	95% CI	
			Low	High
*Cost*				
A	6.377**** (for the linear mixture^c^)	**7.92**^**5**^	-	-
B		**5.96**^**5**^	-	-
C		**6.23**^**5**^	-	-
*Cost savings*^d^				
A	6.377**** (for the linear mixture^c^)	**0.00**	-	-
B		**24.65**	-	-
C		**21.29**	-	-
*Pupation*				
A	8.52*** (for the linear mixture^c^)	**0.83**	0.70	0.96
B		**0.70**	0.57	0.84
C		**0.42**	0.29	0.55
*Duration of the larval stage*				
A	45.63**** (for the linear mixture^c^)	**9.10**	8.47	9.46
B		**10.81**	10.45	11.17
C		**11.30**	10.93	11.66
A×B	0.12^ns^	-0.32	-2.23	1.59
A×C	1.02^ns^	-0.92	-2.77	0.94
B×C	0.50^ns^	-0.70	-2.70	1.31
A^2^×B×C	9.68**	**59.32**	20.43	98.22
A×B^2^×C	7.32*	**-52.62**	-92.27	-12.96
*Pupal weight*				
A	5.00* (for the linear mixture^c^)	**17.98**	16.39	19.56
B		**17.59**	15.84	19.35
C		**15.64**	13.89	17.39
A×C	4.54*	**3.94**	0.39	16.39
B×C	4.25*	**-8.44**	-17.77	-0.12
*Adult emergence*				
A	6.46** (for the linear mixture^c^)	**0.90**	0	0.97
B		**0.83**	0.69	0.93
C		**0.99**	0.96	0.98
B×C	7.07*	**-0.81**	-0.85	-0.07

Significant coefficients appear in bold. The response variables have different number of model terms according to the type of model fitted and to the elimination of non-significant model terms as indicated in Table 1.

^a^ The ANOVA *F* value and the probability that the *F* value occurred due to noise: * P < 0.05; ** P < 0.01; *** P < 0.001; **** P < 0.0001; ns P ≥ 0.05.

^b^ Expressed in terms of coded units by placing their low and high range value between -1 and +1, thus the magnitude of the terms can be compared directly.

^c^ The linear mixture compares the response at the extreme ends of the model, i.e., at the points in the triangle comprising yeast: corn flour: corncob fractions mixtures of: 6: 5.3: 19 (top vertex), 3: 8.3: 19 (left vertex) and 3: 5.3: 22 (right vertex).

^d^ Coefficients are shown for cost savings in %; coefficients for cost savings in US dollars are: A = 0.00, B = 1.95^5^ and C = 1.69^5^.

### Pupation

**Fig 3:**
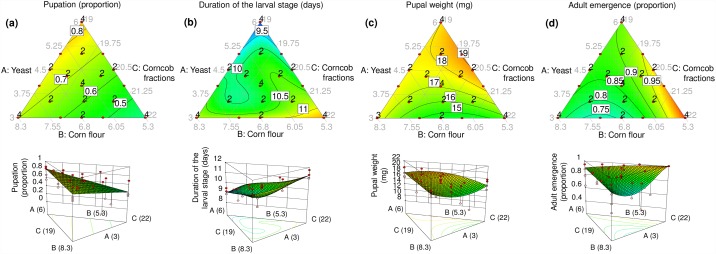
Response surface models fitted to experimental data on (a) pupation (proportion), (b) duration of the larval stage (days), (c) pupal weight (mg) and (d) adult emergence (proportion) of *Anastrepha ludens* as a function of yeast: corn flour: corncob fractions mixtures in the artificial diet. Two-dimensional and three-dimensional plots for each response variable are shown in the upper and lower row, respectively.

The proportion of larvae that pupated ranged from 0.08 to 1.0, with an overall mean (± SE) of 0.65 ± 0.027. A linear model showed that pupation of larval insects increased as a function of the proportion of yeast in the mixture (Tables 2 and 3, Fig 3a). The effect of yeast on pupation was almost double that of corncob fractions, which had the weakest effect of all three ingredients (Table 3).

### Duration of the larval stage

The duration of the larval stage ranged from an average of 8.86 ± 0.19 to 11.78 ± 0.32 days, with an overall mean of 10.29 ± 0.11 days. A reduced special quartic model revealed linear and three-component interaction effects of yeast: corn flour: corncob fractions mixtures on the duration of the larval stage (Tables 2 and 3, Fig 3b). The linear effect of corncob fractions contributed most to increase the duration of the larval stage, followed by corn flour and yeast (Table 3, Fig 3b). Interactive effects among the three ingredients were positive and negative (coefficient estimates for A^2^×B×C and A×B^2^×C in Table 3). These interactions are apparent in the upward and downward curves of the 3-D response surface model across the yeast and corn flour edges (Fig 3b).

### Pupal weight

The mean weight of 3 day-old pupae ranged from 9.22 ± 0.95 to 20.53 ± 0.39 mg, with an overall mean of 17.05 ± 0.36 mg. A reduced quadratic model revealed linear and two-component interaction effects (Tables 2 and 3, Fig 3c). Yeast had the strongest positive linear effect on pupal weight, followed by corn flour, whereas corncob fractions had the weakest effect (Table 3, Fig 3c). Interactive effects among mixture components affected pupal weight positively and negatively. A significant reduction in pupal weight was detected across corn flour: corncob fractions mixtures (negative coefficient on B×C, Table 3). The positive coefficient on A×C (Table 3), indicated a significant increase of pupal weight across the corncob fractions edge. This is illustrated by the downward and upward curves of the 3-D response surface model (Fig 3c).

### Adult emergence

The proportion of adult emergence ranged from 0.5 to 1.0, with an overall mean of 0.86 (± 0.021). A reduced quadratic model showed linear and two-component interaction effects of yeast: corn flour: corncob fractions mixtures on adult emergence (Tables 2 and 3, Fig 3d). Corncob fractions had the largest positive linear coefficient in the model, followed by yeast and corn flour (Table 3). The response surface of adult emergence went down and up across mixtures of corn flour and corncob fractions (Fig 3d).

### Graphical optimization

**Fig 4:**
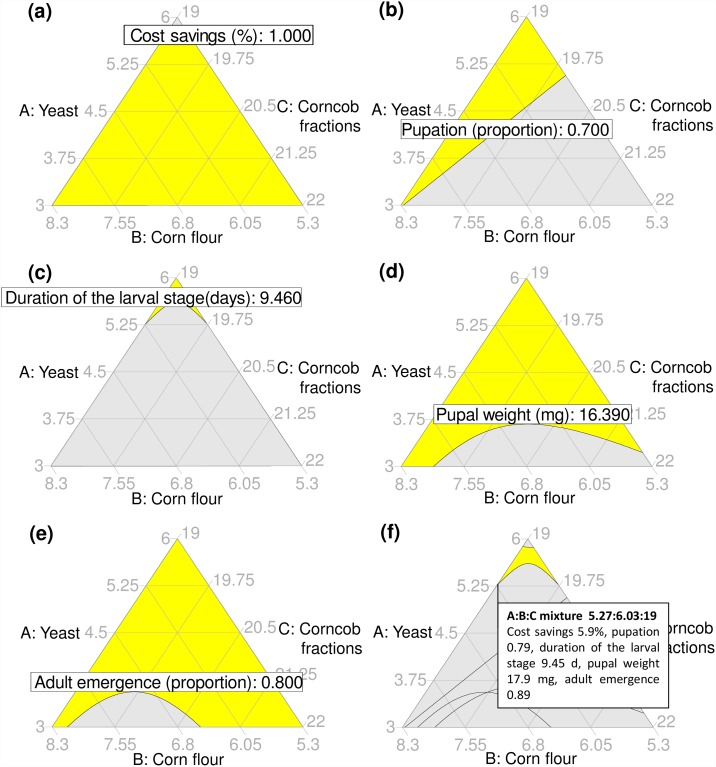
Graphical optimization. (a) Annual cost savings, (b) pupation, (c) duration of the larval stage, (d) pupal weight, (e) adult emergence and (f) overlay contour plot depicting the area of operability. That is, the experimental area meeting all optimization criteria, in which the predicted responses of *A*. *ludens* flies to the yeast: corn flour: corncob fractions mixtures representing the highest savings of all mixtures in the area of operability is shown. The yellow shaded area in each plot indicates the experimental space meeting optimization criteria, the gray shaded area is the space that does not fit those criteria.

The minimum acceptable limits in the optimization models for pupation, pupal weight and adult emergence were 0.7 (proportion), 16.39 (mg) and 0.8 (proportion), respectively; whereas the maximum acceptable limit for the duration of the larval stage was 9.46 days (see 95% CI estimated for term A in Table 3). Except for the duration of the larval stage, models fitted to all other response variables had a relatively wide area meeting optimization criteria (shown as the yellow shaded area in Fig 4a–4e). The duration of the larval stage was most influential in limiting the size of the area of operability in the overlay optimization plot (Fig 4f). The area of operability was located within a range of 5.27% ≤ yeast ≤ 5.88%, 5.42% ≤ corn flour ≤ 6.03%, 19% ≤ corncob fractions ≤ 19.71% (Fig 4f). The maximum annual savings predicted by the model was 5.9% compared to the annual cost of the standard mass rearing mixture, representing savings of US$47,496 for the annual production of 1,730.29 tons of diet at the Moscafrut mass rearing facility (Fig 4f).

## Discussion

We used a mixture experiment and response surface methods to show that a modest reduction in the proportion of yeast in mixtures with corn flour and corncob fractions in the artificial diet of *A*. *ludens*, can provide important savings in diet costs, without apparent reduction in a range of quality indicators of insects produced for SIT. Specifically, our models revealed an experimental design space constrained by 5.27% ≤ yeast ≤ 5.88%, 5.42% ≤ corn flour ≤ 6.03%, 19% ≤ corncob fractions ≤ 19.71% in the diet of *A*. *ludens*, that could generate a similar number of flies with similar larval development times and pupal weights to those produced on a standard diet formulation (Fig 4f). Annual savings of using mixtures proposed by our models, could be up to 5.9% of the total cost of the yeast: corn flour: corncob fractions mixture used in the standard mass rearing diet (Fig 4f). This represents a potential saving of US $27.45 per ton of diet, equivalent to US $47,496 for the mean annual production of 1,730.29 tons of artificial diet in the Moscafrut mass rearing facility. These findings support our predictions and the working hypothesis that yeast level in the diet of *A*. *ludens* for use in SIT can be reduced to improve cost-effectiveness of fly production.

Our laboratory study provides the basis for a detailed mass rearing level study aimed at reducing *A*. *ludens* artificial diet costs. To this end, the area of operability revealed by our models (Fig 4f) could be augmented to create an experimental space including a standard/control mixture (6% yeast, 5.3% corn flour and 19% corncob fractions in this study). The experimental space could be also augmented to yeast proportions below 5.2% to increase cost savings. However, this could result in longer larval development times (Fig 4c), delaying schedule at the mass rearing facility level. It might be worth using a degree-day approach to calculate if longer development times in such diets could be offset by a small increase in rearing temperature. In fact, high metabolic heat produced by high tephritid larval densities in artificial diet is known to affect developmental rates [34]. The larval density used in our study (1 larvae/g of diet) is lower than that used in Moscafrut (3.8–4.8 larvae/g of diet) [35]. Therefore, larval density should be considered as a predictor variable in future studies on *A*. *ludens* artificial diet optimization. Experiments under mass rearing conditions will require examination of several quality control parameters following standard methods established for the mass production of tephritid pests [36]. This should include assessing flight ability, starvation resistance and male sexual competitiveness [36–38]. Before a change in the diet for mass production of *A*. *ludens* could be made, it will also be necessary to evaluate the adaptation of flies to dietary changes across several generations [39, 40].

Previous studies have contributed to the refinement of *A*. *ludens* artificial diet from its original formula based on carrot flour as the main bulking agent and source of vitamins [41]. Searching for efficient and more economical diets has long been a focus of research in this field [23, 42]. The mixture experimental design used here provide a unique tool for artificial diet optimization, and is more suited than one-variable-at-a-time or full factorial designs for studying artificial diets with multiple ingredients [10, 13, 16]. An advantage of mixture experiments is the ability to examine synergistic and antagonistic interactions [2, 15, 16]. Indeed, our models indicate that mixtures of yeast: corn flour: corncob fractions in the artificial diet of *A*. *ludens* can mix antagonistically and synergistically affecting *A*. *ludens* larval development time, pupal weight and adult emergence. These interactions are manifested by the quadratic and quartic models revealing responses of higher or lower magnitude than would be expected from simply adding the effects of each ingredient alone (see coefficient estimates in Table 3).

As the standard *A*. *ludens* artificial diet can be used as a generic diet for rearing other *Anastrepha* species [43], diets arising from our models could also be used for mass rearing other pestiferous fruit fly species worldwide. Tephritid mass rearing facilities around the world could benefit from the application of the experimental and modeling approach reported here for diet optimization. This approach should also be useful for the examination of dietary requirements and for the development of new artificial diet formulations for species that have proven difficult to mass rear on artificial diet (e.g., [44]). Certainly, mixture experiments and RSM modeling approaches are particularly suited to the optimization of biological characteristics of a broad variety of artificially-reared animals or to reducing costs of artificial diets used for rearing [10, 13, 16, 45, 46].

We showed the usefulness of mixture experiments and RSM approaches to model the cost-effectiveness of insect rearing on artificial diets. We hope that in the future, more artificial diet researchers adopt these methods. Managers of other types of animal food production facilities aimed at dogs, cats, shrimp, fish, cattle or chickens could also benefit from this type of approach. Formalizing standard methods among artificial diet researchers should help rearing science and technology to become a formal scientific subdiscipline [47]. We conclude that, although further studies based on our models are needed at the rearing facility level to determine whether yeast-reduced artificial diets can be used effectively for mass rearing of *A*. *ludens* for use in SIT, our approach was proven suitable as a method for the rapid identification of areas of opportunity in diet optimization, and for reducing diet-associated costs.

## Supporting information

S1 Appendix Models fitted to data on the duration of the larval stage and pupal weight including runs 39 and 23, respectively. (DOCX)

S1 Table Experimental design layout and recorded response variables of a mixture experiment aimed to model the cost-effectiveness of rearing *A*. *ludens* on artificial diet. (XLSX)

S2 Table Calculations of the costs and cost savings of the yeast: corn flour: corncob fractions mixtures tested. (XLSX)
