# Supplementary material for: Modeling the cost-effectiveness of insect rearing on artificial diets: A test with a tephritid fly used in the sterile insect technique
Source: PLoS One. 2017 Mar 3;12(3):e0173205. doi: 10.1371/journal.pone.0173205 (PMC5336270; doi:10.1371/journal.pone.0173205)
Supplement: S1 Appendix — (DOCX) [file pone.0173205.s001.docx]

**S1 Appendix**

**Models fitted to data on the duration of the larval stage and pupal weight including runs 39 and 23, respectively.**

**Duration of the larval stage (days)**

**Table A. Anova and summary statistics of a reduced special quartic model fitted to data on the duration of the larval stage (days^-2.18^) including run 39.** Transformation (days^-2.18^) was suggested by a Box-Cox plot analysis of a reduced cubic model fitted first to untransformed data. Yeast (A), corn flour (B) and corncob fractions (C).

| **Analysis of variance table [Partial sum of squares - Type III]** | | | | | | |
| --- | --- | --- | --- | --- | --- | --- |
|  | **Sum of** |  | **Mean** | **F** | **p-value** |  |
| **Source** | **Squares** | **df** | **Square** | **Value** | **Prob > F** |  |
| Model | 3.215E-5 | 7 | 4.592E-6 | 12.90 | < 0.0001 |  |
| Linear Mixture | 2.832E-5 | 2 | 1.416E-5 | 39.79 | < 0.0001 |  |
| AB | 1.771E-8 | 1 | 1.771E-8 | 0.050 | 0.8249 |  |
| AC | 6.136E-8 | 1 | 6.136E-8 | 0.17 | 0.6808 |  |
| BC | 1.736E-7 | 1 | 1.736E-7 | 0.49 | 0.4900 |  |
| A^2^BC | 2.855E-6 | 1 | 2.855E-6 | 8.02 | 0.0079 |  |
| AB^2^C | 3.293E-6 | 1 | 3.293E-6 | 9.25 | 0.0047 |  |
| Residual | 1.139E-5 | 32 | 3.560E-7 |  |  |  |
| Lack of Fit | 6.815E-6 | 11 | 6.196E-7 | 2.84 | 0.0190 |  |
| Pure Error | 4.575E-6 | 21 | 2.179E-7 |  |  |  |
| Cor Total | 4.354E-5 | 39 |  |  |  |  |

|  | R-Squared | 0.7384 |
| --- | --- | --- |
|  | Adj R-Squared | 0.6811 |
|  | Pred R-Squared | 0.5741 |
|  | Adeq Precision | 11.785 |

**Table B. Estimated model coefficients and 95% confidence intervals of a reduced special quartic model fitted to data on the duration of the larval stage (days^-2.18^) including run 39.**

|  | **Coefficient** | **95% CI** | **95% CI** |
| --- | --- | --- | --- |
| **Component** | **Estimate** | **Low** | **High** |
| A-Yeast | 8.128E-3 | 7.536E-3 | 8.721E-3 |
| B-Corn flour | 5.661E-3 | 5.069E-3 | 6.253E-3 |
| C-Corncob fractions | 4.992E-3 | 4.400E-3 | 5.584E-3 |
| AB | -3.407E-4 | -3.452E-3 | 2.771E-3 |
| AC | 6.128E-4 | -2.394E-3 | 3.619E-3 |
| BC | -1.041E-3 | -4.078E-3 | 1.996E-3 |
| A^2^BC | -0.088 | -0.15 | -0.025 |
| AB^2^C | 0.094 | 0.031 | 0.16 |

**Table C. Statistic values from diagnostic plots of a reduced special quartic model fitted to data on the duration of the larval stage (days^-2.18^) including run 39.** Values in red exceed the limits of a particular diagnostic.

|  |  |  |  |  | **Internally** | **Externally** | **Influence on** |  |  |
| --- | --- | --- | --- | --- | --- | --- | --- | --- | --- |
| **Run** | **Actual** | **Predicted** |  |  | **Studentized** | **Studentized** | **Fitted Value** | **Cook's** |  |
| **Order** | **Value** | **Value** | **Residual** | **Leverage** | **Residual** | **Residual** | **DFFITS** | **Distance** |  |
| 1 | 6.808E-3 | 6.402E-3 | 4.057E-4 | 0.206 | 0.763 | 0.758 | 0.387 | 0.019 |  |
| 2 | 8.594E-3 | 8.128E-3 | 4.659E-4 | 0.238 | 0.894 | 0.891 | 0.498 | 0.031 |  |
| 3 | 7.800E-3 | 6.810E-3 | 9.906E-4 | 0.268 | 1.941 | 2.034 | 1.232 | 0.173 |  |
| 4 | 8.608E-3 | 8.128E-3 | 4.797E-4 | 0.238 | 0.921 | 0.918 | 0.513 | 0.033 |  |
| 5 | 6.309E-3 | 6.292E-3 | 1.693E-5 | 0.153 | 0.031 | 0.030 | 0.013 | 0.000 |  |
| 6 | 5.094E-3 | 5.661E-3 | -5.670E-4 | 0.237 | -1.088 | -1.091 | -0.609 | 0.046 |  |
| 7 | 6.729E-3 | 6.713E-3 | 1.514E-5 | 0.248 | 0.029 | 0.029 | 0.017 | 0.000 |  |
| 8 | 5.997E-3 | 6.256E-3 | -2.590E-4 | 0.113 | -0.461 | -0.455 | -0.163 | 0.003 |  |
| 9 | 4.625E-3 | 4.992E-3 | -3.675E-4 | 0.237 | -0.705 | -0.700 | -0.390 | 0.019 |  |
| 10 | 7.987E-3 | 6.719E-3 | 1.268E-3 | 0.214 | 2.398 | 2.605 | 1.361 | 0.196 |  |
| 11 | 6.126E-3 | 6.810E-3 | -6.838E-4 | 0.268 | -1.340 | -1.358 | -0.822 | 0.082 |  |
| 12 | 5.305E-3 | 4.992E-3 | 3.132E-4 | 0.237 | 0.601 | 0.595 | 0.332 | 0.014 |  |
| 13 | 6.673E-3 | 6.402E-3 | 2.709E-4 | 0.206 | 0.510 | 0.504 | 0.257 | 0.008 |  |
| 14 | 6.261E-3 | 5.837E-3 | 4.235E-4 | 0.163 | 0.776 | 0.771 | 0.340 | 0.015 |  |
| 15 | 6.151E-3 | 6.525E-3 | -3.742E-4 | 0.130 | -0.672 | -0.666 | -0.257 | 0.008 |  |
| 16 | 5.367E-3 | 4.992E-3 | 3.752E-4 | 0.237 | 0.720 | 0.715 | 0.398 | 0.020 |  |

**Table C.** *Continued*.

|  |  |  |  |  | **Internally** | **Externally** | **Influence on** |  |  |
| --- | --- | --- | --- | --- | --- | --- | --- | --- | --- |
| **Run** | **Actual** | **Predicted** |  |  | **Studentized** | **Studentized** | **Fitted Value** | **Cook's** |  |
| **Order** | **Value** | **Value** | **Residual** | **Leverage** | **Residual** | **Residual** | **DFFITS** | **Distance** |  |
| 17 | 7.038E-3 | 6.713E-3 | 3.245E-4 | 0.248 | 0.627 | 0.621 | 0.356 | 0.016 |  |
| 18 | 6.261E-3 | 5.837E-3 | 4.235E-4 | 0.163 | 0.776 | 0.771 | 0.340 | 0.015 |  |
| 19 | 6.029E-3 | 5.589E-3 | 4.399E-4 | 0.067 | 0.763 | 0.758 | 0.203 | 0.005 |  |
| 20 | 6.286E-3 | 6.292E-3 | -5.202E-6 | 0.153 | -0.009 | -0.009 | -0.004 | 0.000 |  |
| 21 | 5.029E-3 | 4.992E-3 | 3.704E-5 | 0.237 | 0.071 | 0.070 | 0.039 | 0.000 |  |
| 22 | 8.032E-3 | 8.128E-3 | -9.594E-5 | 0.238 | -0.184 | -0.181 | -0.101 | 0.001 |  |
| 23 | 5.684E-3 | 5.661E-3 | 2.302E-5 | 0.237 | 0.044 | 0.043 | 0.024 | 0.000 |  |
| 24 | 5.940E-3 | 5.066E-3 | 8.738E-4 | 0.254 | 1.696 | 1.749 | 1.020 | 0.122 |  |
| 25 | 6.692E-3 | 6.719E-3 | -2.650E-5 | 0.214 | -0.050 | -0.049 | -0.026 | 0.000 |  |
| 26 | 5.973E-3 | 5.589E-3 | 3.834E-4 | 0.067 | 0.665 | 0.659 | 0.176 | 0.004 |  |
| 27 | 6.151E-3 | 6.256E-3 | -1.046E-4 | 0.113 | -0.186 | -0.183 | -0.066 | 0.001 |  |
| 28 | 6.224E-3 | 6.256E-3 | -3.187E-5 | 0.113 | -0.057 | -0.056 | -0.020 | 0.000 |  |
| 29 | 5.819E-3 | 6.256E-3 | -4.369E-4 | 0.113 | -0.778 | -0.773 | -0.276 | 0.010 |  |
| 30 | 5.523E-3 | 5.661E-3 | -1.388E-4 | 0.237 | -0.266 | -0.262 | -0.146 | 0.003 |  |
| 31 | 7.439E-3 | 8.128E-3 | -6.896E-4 | 0.238 | -1.324 | -1.340 | -0.748 | 0.068 |  |
| 32 | 4.872E-3 | 5.066E-3 | -1.948E-4 | 0.254 | -0.378 | -0.373 | -0.217 | 0.006 |  |
| 33 | 5.789E-3 | 5.661E-3 | 1.277E-4 | 0.237 | 0.245 | 0.241 | 0.135 | 0.002 |  |
| 34 | 5.806E-3 | 6.525E-3 | -7.199E-4 | 0.130 | -1.293 | -1.307 | -0.504 | 0.031 |  |
| 35 | 6.607E-3 | 6.408E-3 | 1.990E-4 | 0.224 | 0.379 | 0.374 | 0.201 | 0.005 |  |
| 36 | 6.670E-3 | 7.230E-3 | -5.602E-4 | 0.225 | -1.066 | -1.069 | -0.575 | 0.041 |  |
| 37 | 6.688E-3 | 7.219E-3 | -5.314E-4 | 0.213 | -1.004 | -1.004 | -0.523 | 0.034 |  |
| 38 | 5.470E-3 | 6.174E-3 | -7.036E-4 | 0.204 | -1.322 | -1.338 | -0.678 | 0.056 |  |
| 39 | 3.344E-3 | 4.984E-3 | -1.640E-3 | 0.209 | -3.091 | -3.632 | -1.868 | 0.316 |  |
| 40 | 5.481E-3 | 5.207E-3 | 2.742E-4 | 0.218 | 0.520 | 0.514 | 0.271 | 0.009 |  |

**Figure A. Reduced special quartic model fitted to data on the duration of the larval stage (days^-2.18^) including run 23.**

**Pupal weight (mg)**

**Table D. Anova and summary statistics of a reduced quartic model fitted to data on pupal weight including run 23.** Yeast (A), corn flour (B) and corncob fractions (C).

| **Analysis of variance table [Partial sum of squares - Type III]** | | | | | | |
| --- | --- | --- | --- | --- | --- | --- |
|  | **Sum of** |  | **Mean** | **F** | **p-value** |  |
| **Source** | **Squares** | **df** | **Square** | **Value** | **Prob > F** |  |
| Model | 118.11 | 10 | 11.81 | 4.40 | 0.0008 |  |
| Linear Mixture | 39.77 | 2 | 19.88 | 7.40 | 0.0025 |  |
| AB | 0.58 | 1 | 0.58 | 0.22 | 0.6462 |  |
| AC | 1.37 | 1 | 1.37 | 0.51 | 0.4811 |  |
| BC | 3.69 | 1 | 3.69 | 1.37 | 0.2506 |  |
| BC(B-C) | 23.30 | 1 | 23.30 | 8.68 | 0.0063 |  |
| AB^2^C | 17.90 | 1 | 17.90 | 6.67 | 0.0151 |  |
| ABC^2^ | 35.21 | 1 | 35.21 | 13.11 | 0.0011 |  |
| AB(A-B)^2^ | 5.85 | 1 | 5.85 | 2.18 | 0.1507 |  |
| BC(B-C)^2^ | 15.10 | 1 | 15.10 | 5.62 | 0.0246 |  |
| Residual | 77.89 | 29 | 2.69 |  |  |  |
| Lack of Fit | 5.46 | 8 | 0.68 | 0.20 | 0.9881 |  |
| Pure Error | 72.43 | 21 | 3.45 |  |  |  |
| Cor Total | 196.00 | 39 |  |  |  |  |

|  | R-Squared | 0.6026 |
| --- | --- | --- |
|  | Adj R-Squared | 0.4655 |
|  | Pred R-Squared | 0.2047 |
|  | Adeq Precision | 11.509 |

**Table E. Estimated model coefficients and 95% confidence intervals of a reduced quartic model fitted to data on pupal weight including run 23.**

|  | **Coefficient** | **95% CI** | **95% CI** |
| --- | --- | --- | --- |
| **Component** | **Estimate** | **Low** | **High** |
| A-Yeast | 17.71 | 16.07 | 19.36 |
| B-Corn flour | 16.04 | 14.38 | 17.69 |
| C-Corncob fractions | 16.16 | 14.50 | 17.82 |
| AB | 2.07 | -7.04 | 11.18 |
| AC | 2.92 | -5.44 | 11.27 |
| BC | -5.22 | -14.32 | 3.89 |
| BC(B-C) | 44.22 | 13.52 | 74.93 |
| AB^2^C | -279.95 | -501.72 | -58.18 |
| ABC^2^ | 397.13 | 172.81 | 621.45 |
| AB(A-B)^2^ | 50.60 | -19.51 | 120.72 |
| BC(B-C)^2^ | -78.11 | -145.48 | -10.74 |

**Table F. Statistic values from diagnostic plots of a reduced quartic model fitted to data on pupal weight including run 23.** Values in red exceed the limits of a particular diagnostic.

|  |  |  |  |  | **Internally** | **Externally** | **Influence on** |  |  |
| --- | --- | --- | --- | --- | --- | --- | --- | --- | --- |
| **Run** | **Actual** | **Predicted** |  |  | **Studentized** | **Studentized** | **Fitted Value** |  | **Cook's** |
| **Order** | **Value** | **Value** | **Residual** | **Leverage** | **Residual** | **Residual** | **DFFITS** |  | **Distance** |
| 1 | 18.62 | 19.35 | -0.73 | 0.251 | -0.516 | -0.509 | -0.295 |  | 0.008 |
| 2 | 16.78 | 17.71 | -0.93 | 0.240 | -0.654 | -0.647 | -0.364 |  | 0.012 |
| 3 | 14.56 | 17.39 | -2.83 | 0.340 | -2.128 | -2.276 | -1.633 |  | 0.212 |
| 4 | 16.50 | 17.71 | -1.21 | 0.240 | -0.845 | -0.841 | -0.473 |  | 0.021 |
| 5 | 17.46 | 17.46 | -2.56E-3 | 0.178 | -0.002 | -0.002 | -0.001 |  | 0.000 |
| 6 | 18.35 | 16.04 | 2.32 | 0.245 | 1.626 | 1.676 | 0.954 |  | 0.078 |
| 7 | 16.59 | 17.66 | -1.07 | 0.253 | -0.759 | -0.753 | -0.438 |  | 0.018 |
| 8 | 17.87 | 18.06 | -0.18 | 0.138 | -0.120 | -0.118 | -0.047 |  | 0.000 |
| 9 | 14.23 | 16.16 | -1.93 | 0.245 | -1.352 | -1.372 | -0.782 |  | 0.054 |
| 10 | 14.73 | 15.53 | -0.80 | 0.405 | -0.633 | -0.626 | -0.517 |  | 0.025 |
| 11 | 18.95 | 17.39 | 1.56 | 0.340 | 1.168 | 1.176 | 0.843 |  | 0.064 |
| 12 | 15.58 | 16.16 | -0.58 | 0.245 | -0.405 | -0.399 | -0.227 |  | 0.005 |
| 13 | 20.53 | 19.35 | 1.18 | 0.251 | 0.833 | 0.828 | 0.480 |  | 0.021 |
| 14 | 19.05 | 19.65 | -0.60 | 0.188 | -0.406 | -0.400 | -0.193 |  | 0.003 |
| 15 | 16.75 | 16.10 | 0.65 | 0.231 | 0.455 | 0.449 | 0.247 |  | 0.006 |

**Table F.** *Continued.*

|  |  |  |  |  | **Internally** | **Externally** | **Influence on** |  |  |
| --- | --- | --- | --- | --- | --- | --- | --- | --- | --- |
| **Run** | **Actual** | **Predicted** |  |  | **Studentized** | **Studentized** | **Fitted Value** |  | **Cook's** |
| **Order** | **Value** | **Value** | **Residual** | **Leverage** | **Residual** | **Residual** | **DFFITS** |  | **Distance** |
| 16 | 17.00 | 16.16 | 0.84 | 0.245 | 0.591 | 0.584 | 0.333 |  | 0.010 |
| 17 | 18.52 | 17.66 | 0.85 | 0.253 | 0.601 | 0.594 | 0.346 |  | 0.011 |
| 18 | 19.05 | 19.65 | -0.60 | 0.188 | -0.404 | -0.398 | -0.192 |  | 0.003 |
| 19 | 15.36 | 15.61 | -0.25 | 0.364 | -0.192 | -0.189 | -0.143 |  | 0.002 |
| 20 | 16.59 | 17.46 | -0.88 | 0.178 | -0.589 | -0.582 | -0.271 |  | 0.007 |
| 21 | 17.59 | 16.16 | 1.43 | 0.245 | 1.004 | 1.004 | 0.572 |  | 0.030 |
| 22 | 19.77 | 17.71 | 2.06 | 0.240 | 1.442 | 1.471 | 0.827 |  | 0.060 |
| 23 | 11.76 | 16.04 | -4.27 | 0.245 | -3.001 | -3.551 | -2.020 |  | 0.265 |
| 24 | 17.74 | 14.79 | 2.95 | 0.345 | 2.223 | 2.398 | 1.739 |  | 0.236 |
| 25 | 15.44 | 15.53 | -0.091 | 0.405 | -0.072 | -0.071 | -0.059 |  | 0.000 |
| 26 | 16.97 | 15.61 | 1.36 | 0.364 | 1.041 | 1.043 | 0.789 |  | 0.056 |
| 27 | 19.38 | 18.06 | 1.33 | 0.138 | 0.872 | 0.868 | 0.347 |  | 0.011 |
| 28 | 18.24 | 18.06 | 0.18 | 0.138 | 0.119 | 0.117 | 0.047 |  | 0.000 |
| 29 | 17.17 | 18.06 | -0.89 | 0.138 | -0.583 | -0.577 | -0.230 |  | 0.005 |
| 30 | 17.39 | 16.04 | 1.35 | 0.245 | 0.948 | 0.947 | 0.539 |  | 0.026 |
| 31 | 17.88 | 17.71 | 0.16 | 0.240 | 0.115 | 0.113 | 0.064 |  | 0.000 |
| 32 | 12.20 | 14.79 | -2.59 | 0.345 | -1.955 | -2.061 | -1.495 |  | 0.183 |
| 33 | 16.68 | 16.04 | 0.64 | 0.245 | 0.453 | 0.446 | 0.254 |  | 0.006 |
| 34 | 16.73 | 16.10 | 0.64 | 0.231 | 0.444 | 0.438 | 0.240 |  | 0.005 |
| 35 | 19.08 | 18.30 | 0.77 | 0.286 | 0.558 | 0.552 | 0.349 |  | 0.011 |
| 36 | 18.96 | 18.86 | 0.094 | 0.270 | 0.067 | 0.066 | 0.040 |  | 0.000 |
| 37 | 17.51 | 17.84 | -0.33 | 0.211 | -0.227 | -0.223 | -0.115 |  | 0.001 |
| 38 | 17.90 | 17.32 | 0.57 | 0.216 | 0.393 | 0.387 | 0.204 |  | 0.004 |
| 39 | 9.22 | 9.75 | -0.53 | 0.697^2^ | -0.586 | -0.580 | -0.879 |  | 0.072 |
| 40 | 16.62 | 16.27 | 0.35 | 0.741^2^ | 0.422 | 0.416 | 0.703 |  | 0.046 |

**Figure B. Reduced quartic model fitted to data on pupal weight including run 23.**
